# Dynamic Changes in Reactive Oxygen Species in the Shoot Apex Contribute to Stem Cell Death in *Arabidopsis thaliana*

**DOI:** 10.3390/ijms23073864

**Published:** 2022-03-31

**Authors:** Yukun Wang, Makoto Shirakawa, Toshiro Ito

**Affiliations:** 1 Henry Fok School of Biology and Agriculture, Shaoguan University, Shaoguan 512000, China; wangyu_kun1@163.com; 2 Graduate School of Biological Science, Nara Institute of Science and Technology, Ikoma 630-0192, Japan

**Keywords:** reactive oxygen species (ROS), superoxide anion ($O_{2}^{\cdot -}$), hydrogen peroxide (H_2_O_2_), shoot stem cell, programmed cell death (PCD), longevity

## Abstract

In monocarpic plants, stem cells are fated to die. However, the potential mechanism of stem cell death has remained elusive. Here, we reveal that the levels of two forms of reactive oxygen species (ROS), superoxide anion free radical ($O_{2}^{\cdot -}$) and hydrogen peroxide (H_2_O_2_), show dynamic changes in the shoot apex during the plant life cycle of *Arabidopsis thaliana*. We found that the level of $O_{2}^{\cdot -}$ decreased and disappeared at four weeks after bolting (WAB), while H_2_O_2_ appeared at 3 WAB and showed a burst at 5 WAB. The timing of dynamic changes in $O_{2}^{\cdot -}$ and H_2_O_2_ was delayed for approximately three weeks in *clv3-2*, which has a longer lifespan. Moreover, exogenous application of H_2_O_2_ inhibited the expression of the stem cell determinant *WUSCHEL* (*WUS*) and promoted the expression of the developmentally programmed cell death (dPCD) marker gene *ORESARA 1* (*ORE1*). These results indicate that H_2_O_2_ triggers an important signal inducing dPCD in stem cells. Given that $O_{2}^{\cdot -}$ plays roles in maintaining *WUS* expression and stem cell activity, we speculate that the dynamic shift from $O_{2}^{\cdot -}$ to H_2_O_2_ in the shoot apex results in stem cell death. Our findings provide novel insights for understanding ROS-mediated regulation during plant stem cell death.

## 1. Introduction

Plant stem cells are harbored in shoot apical meristems (SAMs), which play essential roles in growth and development. Over the past decade, multiple works have shown the molecular mechanisms of stem cell maintenance [1,2]. In *Arabidopsis thaliana*, WUSCHEL (WUS) and CLAVATA3 (CLV3) constitute a negative feedback loop to maintain the homeostasis of the stem cell population [3,4]. *WUS*, as a stem cell marker, not only positively controls meristem activity but also maintains the stem cell population during the postembryonic development stage [5,6]. Several factors, such as the peptide CLV3 and receptor kinases CLV1, CLV2, BARELY ANY MERISTEM (BAM), and receptor-like protein kinase 2 (RPK2)/TOADSTOOL 2, are involved in regulating the spatiotemporal expression pattern of *WUS* in the SAM [7]. As an intracellular signal, the plant hormone cytokinin can also regulate *WUS* expression via both CLV-dependent and CLV-independent pathways [7,8,9]. Recently, the intracellular molecule reactive oxygen species (ROS) have been reported to be involved in regulating stem cell maintenance during the vegetative stage, indicating that ROS balance plays a key role in stem cell fate regulation [10]. ROS, mainly composed of superoxide anion free radical ($O_{2}^{\cdot -}$) and hydrogen peroxide (H_2_O_2_), are naturally produced in all living cells [10]. ROS are associated with stress stimuli such as high light, salinity, heavy metals, cold, and pathogens [11].

As one of the ROS species, H_2_O_2_ has been shown to be a crucial signaling molecule in various growth and developmental processes, including seed germination, leaf development and senescence, development of trichome and male sex organs, development of pollen tubes on the pistil, lateral root and root hair development, aerenchyma formation, and even self-incompatibility [12]. It is worth noting that H_2_O_2_ gives rise to programmed cell death (PCD) in most of the biological processes mentioned above [1,12]. In addition, intracellular redox status is connected to meristem activity in both the vegetative SAM and the root apical meristem (RAM) [10,13,14,15,16]. However, whether ROS influence stem cell status in the senescent SAM during the plant life cycle is still unclear.

We previously observed the senescence phenomenon in shoot stem cells and specified that the final fate of shoot stem cells undergoes a developmental PCD (dPCD) process [2]. In addition, we pointed out that shoot stem cell dPCD is associated with the expression of ROS-related genes [2]. Given that stem cells directly contribute to plant lifespan and that the death of stem cells is an important part of stem cell fate, it is necessary to investigate the ROS-mediated PCD process during the stem cell lifespan more deeply.

In this study, we first monitored the dynamic spatiotemporal patterns of ROS species $O_{2}^{\cdot -}$ and H_2_O_2_ in proliferative and senescent SAMs. By applying exogenous H_2_O_2_, we found that the accumulation of H_2_O_2_ could not only inhibit *WUS* expression but also induce dPCD in stem cells. Moreover, we used two scavengers, N,N′-dimethylthiourea (DMTU) [10,17] and potassium iodide (KI) [10,13], to reduce the levels of $O_{2}^{\cdot -}$ and H_2_O_2_, respectively, in the inflorescence meristem (IM) domain. The results further explored the function of H_2_O_2_ in shutting down stem cell activity, leading to the activation of the dPCD process in the stem cell population. Based on this evidence, we established a regulatory model in which ROS dynamics were thought to be at the core of explaining the ROS-mediated mechanism of stem cell dPCD. The results of this study can enrich our understanding of age-dependent dPCD in stem cells and may help to facilitate the regulatory mechanism of stem cell fate governing the whole plant lifespan.

## 2. Results

### 2.1. Dynamic Changes in ROS Components in Wild-Type and clv3-2 Mutant Plants

Our previous study revealed that the inflorescence meristem (IM) of *clv3-2* mutants possesses a longer longevity. Moreover, *clv3-2* produces an increased number of flowers and siliques on primary shoots (Figure S1) [2]. Thus, we think that CLV3 may be a safeguard that prevents the longer expression time of *WUS* [2]. However, we found that the expression of *WUS* was still terminated at 6 weeks after bolting (WAB) in *clv3-2* mutants (Figure S2) [2], indicating that there must be other factors that inhibit *WUS* transcription. Recently, a study reported that two ROS components, $O_{2}^{\cdot -}$ and H_2_O_2_, are involved in regulating stem cell development and that the balance between them is indispensable to stem cell maintenance and differentiation [10]. Moreover, $O_{2}^{\cdot -}$ can activate *WUS* expression and maintain stemness, and H_2_O_2_ accumulation in the peripheral zone (PZ) negatively regulates $O_{2}^{\cdot -}$ biosynthesis, resulting in stem cell termination [10]. This evidence strongly suggests that ROS homeostasis influences stem cell fate determination and that H_2_O_2_ may be a regulator of *WUS* expression. More significantly, our preceding work also shows that the H_2_O_2_ signal may be involved in *WUS* expression termination [2]. Therefore, it is necessary to understand the spatial-temporal patterns of ROS components in the IM.

**Figure 1:**
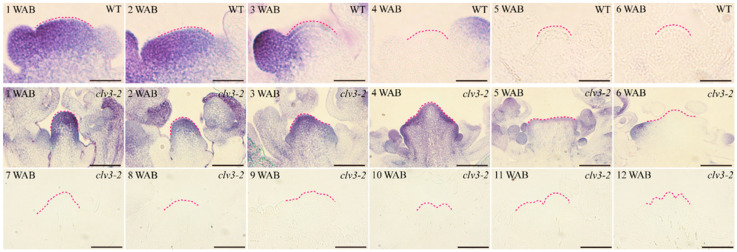
NBT staining of WT and *clv3-2* IMs to demonstrate the $O_{2}^{\cdot -}$ signals (blue color). Dotted pink lines indicate the IM shape. Scale bars: 25 μm in WT and 100 μm in *clv3-2*.

Through NBT (4-nitro blue tetrazolium chloride) staining, we examined the distribution and accumulation characteristics of $O_{2}^{\cdot -}$ in the IM domains of the WT and the *clv3-2* mutant. In WT, $O_{2}^{\cdot -}$ displayed a strong signal in the IM domain, especially in the stem cell layers, at 1 WAB and 2 WAB. At 3 WAB, the level of $O_{2}^{\cdot -}$ in the stem cells was clearly decreased in the IM (pink dashed lines) but became undetectable from 4 WAB to 6 WAB (Figure 1). In the *clv3-2* mutant, $O_{2}^{\cdot -}$ was detected for 2 weeks longer than in the WT (from 1 WAB to 5 WAB). Clear $O_{2}^{\cdot -}$ signals were obviously displayed in the *clv3-2* IM domain from 1 WAB to 4 WAB. At 5 WAB, the level of $O_{2}^{\cdot -}$ in the stem cell layers was reduced and fully disappeared from the stem cells from 6 WAB (Figure 1).

**Figure 2:**
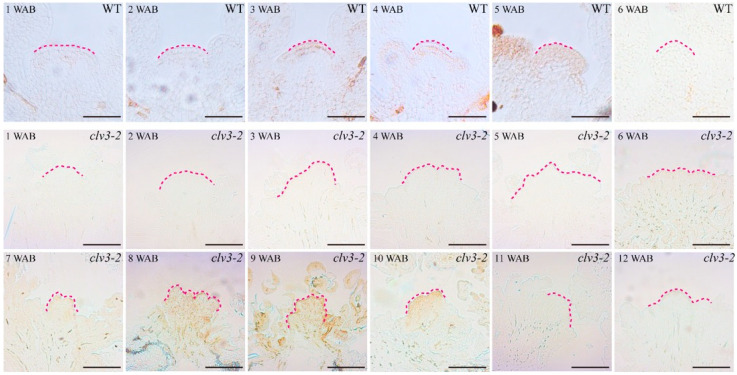
DAB staining of WT and *clv3-2* IMs to demonstrate the H_2_O_2_ signals (brown color). Dotted pink lines indicate the IM shape. Scale bars: 25 μm in WT and 100 μm in *clv3-2*.

The spatial-temporal distribution of H_2_O_2_ in the WT and *clv3-2* IM domains was observed using DAB (3,3′-diaminobenzidine) staining. In the WT IM domain, H_2_O_2_ initially appeared at 3 WAB and showed a burst at 5 WAB (Figure 2). There were no detectable signals in *clv3-2* IM domains from 1 WAB to 5 WAB. Then, H_2_O_2_ signals initially appeared in the *clv3-2* IM domain at 6 WAB. Subsequently, clear and increased H_2_O_2_ signals were detected in the *clv3-2* IM domain until 10 WAB. Notably, the level of H_2_O_2_ showed an accumulated peak at 10 WAB. At 11 and 12 WABs, there were no detectable H_2_O_2_ signals due to whole IM death (Figure 2 and Figure S1). Compared with the WT, H_2_O_2_ signals appeared 3 weeks later in the *clv3-2* IM domain (Figure 2).

Based on these findings, we found that the time during which $O_{2}^{\cdot -}$ and H_2_O_2_ were detected displayed a switch period. In WT, this switch period occurred from 3 WAB to 4 WAB, and the level of $O_{2}^{\cdot -}$ decreased from 3 WAB and fully disappeared from the IM domain at 4 WAB. In addition, a very weak H_2_O_2_ signal was initially detected in the stem cell layers at 3 WAB and became stronger at 4 WAB. Similarly, there was also a conversion period of the time during which $O_{2}^{\cdot -}$ and H_2_O_2_ were detected in *clv3-2* mutants from 6 WAB to 7 WAB, which represented a two-week delay compared with that in WT (Figure 1 and Figure 2). These results indicated that dynamic changes in ROS components occurred during the lifespan of IM in both WT and *clv3-2* mutants, and the deferred conversion period in *clv3-2* IM was in line with the prolonged *WUS* expression time, suggesting that ROS conversion might regulate the expression time of *WUS*.

### 2.2. Features of Programmed Stem Cell Death in the clv3-2 Mutant

**Figure 3:**
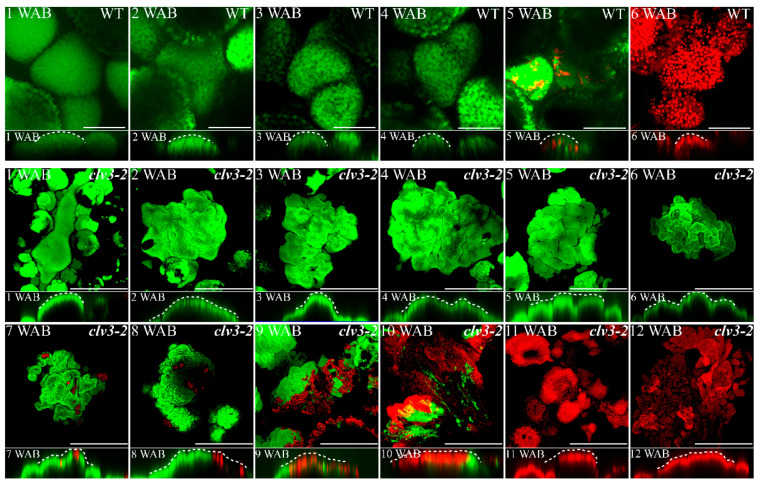
FDA/PI staining of IMs in WT and *clv3-2* mutants. The top view of the confocal images of the FDA (green) and PI (red) signals is shown in the upper panel. The side view is shown in the lower panel. FDA-stained cells (in green) are alive, and PI-stained cells (in red) are dead. White dotted lines indicate IM shapes. Scale bars: 50 μm in WT and 100 μm in *clv3-2* mutants.

Whereas the stem cell death process has been studied by fluorescein diacetate (FDA) and propidium iodide (PI) staining and the key dPCD marker gene *BIFUNCTIONAL NUCLEASE1* (*BFN1*) in WT Arabidopsis [2], similar studies in *clv3-2* stem cells are still lacking. Thus, we examined the details of stem cell death in both WT and *clv3-2* mutants in this study. The FDA/PI staining results showed that stem cell death started at 7 WAB in *clv3-2* IM, and strong PI signals could be detected up to 10 WAB. Subsequently, all stem cells were stained by PI at 11 WAB and 12 WAB, as the entire *clv3-2* IM was completely dead (Figure 3 and Figure S1). In contrast, the PI signal was visible at 5 WAB in WT IM. At 6 WAB, all stem cells were stained by PI because the WT IM was dead (Figure 3 and Figure S1).

**Figure 4:**
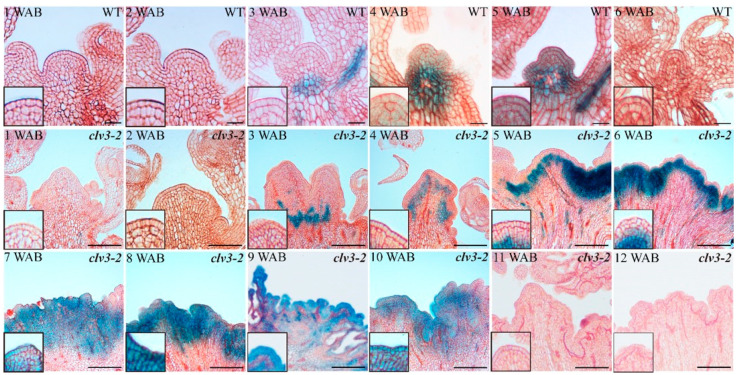
The spatial-temporal expression patterns of *BFN1* in WT and *clv3-2* mutants. The magnified images at the bottom left corner indicate the *BFN1* expression signals (blue color) in stem cell layers. Scale bars: 15 μm in WT and 100 μm in *clv3-2*.

To understand which type of cell death occurred in the dead stem cells, we checked the spatial-temporal expression profiles of dPCD marker genes. The key dPCD marker gene *BFN1*, which encodes a nuclease, was initially expressed in the bottom regions of the *clv3-2* IM domain at 3 WAB. Then, the expression area of *BFN1* moved upward but still did not enter into the stem cell layers until 6 WAB, which is the time point at which *WUS* started to be terminated (Figure 4 and Figure S2). From 7 WAB to 10 WAB, *BFN1* was expressed in stem cells, resulting in stem cell death in *clv3-2* IM (Figure 3 and Figure 4). Because the whole *clv3-2* IM was dead after 11 WAB, there were no *BFN1* signals in the IM region (Figure 4).

**Figure 5:**
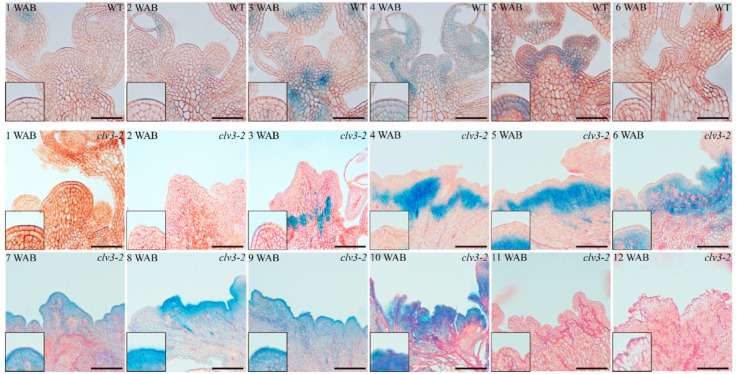
The spatial-temporal expression patterns of *ORE1* in WT and *clv3-2* mutants. The magnified images at the bottom left corner indicate the *ORE1* expression signals (blue color) in the stem cell layers. Scale bars: 50 μm in WT and 100 μm in *clv3-2*.

It has been reported that the NAC transcription factor ORESARA1 (ORE1) directly regulates *BFN1* and that the expression patterns of these two senescence-enhanced genes largely overlap during leaf senescence [18]. However, the spatial-temporal expression profiles of *ORE1* during IM senescence are still unknown. As shown in Figure 5, there were no *ORE1* expression signals (blue color) in the IM domains in WT and *clv3-2* mutants from 1 WAB to 2 WAB. At 3 WAB, *ORE1* was expressed in the bottom area of the IM domain in both WT and *clv3-2* mutants. In WT, the expression domain of *ORE1* moved upward at 4 WAB and showed clear signals in stem cell layers at 5 WAB. The expression signal of *ORE1* could not be detected at 6 WAB because the whole WT IM was dead (Figure 5 and Figure S1). In contrast, in *clv3-2* mutants, the expression region of *ORE1* started to be observed in the upper layers from 4 WAB to 6 WAB. From 7 WAB to 10 WAB, *ORE1* expression signals could be detected in stem cell layers. Because the whole *clv3-2* IM was dead, no *ORE1* expression signals could be detected at 11 WAB or 12 WAB (Figure 5 and Figure S1). These results proved that the spatial-temporal expression profiles of *BFN1* and *ORE1* were almost consistent during the IM senescent period in WT and *clv3-2* mutants and suggested that the *ORE1*-*BFN1* cascade might be involved in programmed stem cell death regulation.

### 2.3. Application of Exogenous H_2_O_2_ Influences WUS and ORE1 Expression

**Figure 6:**
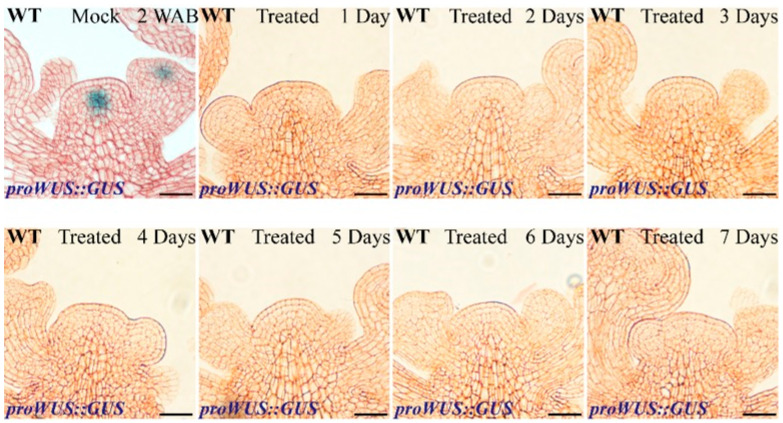
Effects of exogenous H_2_O_2_ on *WUS* expression. The IMs at 1 WAB were treated with 5 mM H_2_O_2_ until 2 WAB. The *WUS* expression profile from 1 day to 7 days is shown. Scale bars: 20 μm.

Based on our previously published results [2] and the abovementioned evidence (Figure 1, Figure 2, Figure 3, Figure 4 and Figure 5), we speculated that the conversion of ROS components in the IM domain governs stem cell activity termination and stem cell senescence and death. To verify this hypothesis, an exogenous H_2_O_2_ assay was performed. First, we studied the effects of exogenous H_2_O_2_ on *WUS* expression under different concentrations and found that 5 mM, 10 mM, and 20 mM H_2_O_2_ could inhibit *WUS* expression after one week of continuous treatment (Figure 6 and Figure S3). This result indicated that 5 mM exogenous H_2_O_2_ treatment was sufficient to terminate *WUS* expression. Next, we studied how soon *WUS* expression responds to 5 mM exogenous H_2_O_2_ treatment. As shown in Figure 6, *WUS* could respond to exogenous H_2_O_2_ after only 1 day of treatment. These results revealed that *WUS* was sensitive to exogenous H_2_O_2_.

**Figure 7:**
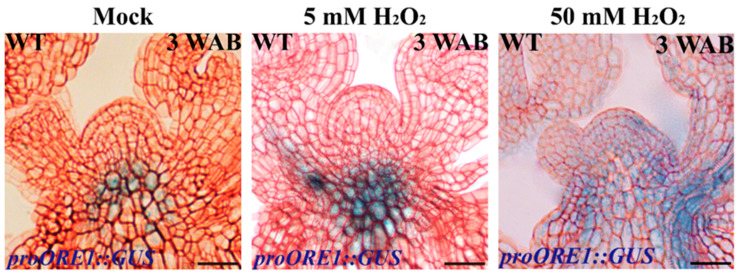
Effects of exogenous H_2_O_2_ on dPCD marker gene *ORE1* expression. The IMs at 2 WAB were treated with 5 mM (negative control) and 50 mM H_2_O_2_ until 3 WAB. The images show GUS signals of *ORE1* at 3 WAB. Scale bars: 20 μm.

In addition, the *proORE1::GUS* line was treated for one week with exogenous H_2_O_2_. After 5, 20, and 40 mM H_2_O_2_ treatments, the expression area of *ORE1* was similar to that in the control group (mock), indicating that *ORE1* did not respond to low concentrations of H_2_O_2_ (Figure 7 and Figure S4). Interestingly, the expression signals of *ORE1* could be detected in stem cell layers after one week of 50 mM H_2_O_2_ treatment (Figure 7). These results revealed that *WUS* inhibition and *ORE1* induction by H_2_O_2_ showed different threshold values.

### 2.4. Effects of Endogenous $O_{2}^{\cdot -}$ and H_2_O_2_ Scavengers

**Figure 8:**
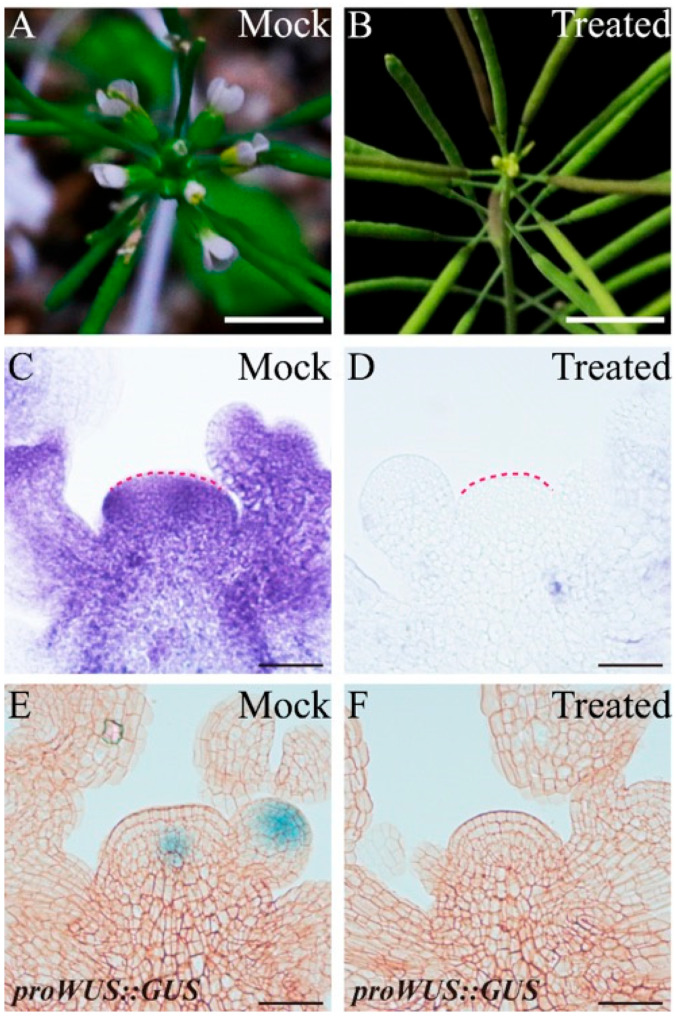
The phenotype of primary shoot inflorescence (**A**,**B**), $O_{2}^{\cdot -}$ accumulation (**C**,**D**), and *WUS* expression pattern (**E**,**F**) of the control group (mock) and 20 mM DMTU-treated group. Pink dotted lines indicate the shape of the IM. Scale bars: 1 cm in (**A**,**B**), 25 μm in (**C**–**F**).

To confirm whether endogenous ROS can regulate *WUS* expression during the proliferative stage, we used the ROS scavengers N,N′-dimethylthiourea (DMTU) and potassium iodide (KI) to reduce endogenous $O_{2}^{\cdot -}$ and H_2_O_2_, respectively. After one week of 20 mM DMTU treatment (the treatment was at 1 WAB), the inflorescence at 2 WAB displayed a clear flowering-arrested phenotype, while several flowers bloomed normally in the control group (mock) (Figure 8A,B). To determine whether the scavenger DMTU effectively inhibits $O_{2}^{\cdot -}$ production in the WT IM domain, we carried out NBT staining of IM at 2 WAB. The results showed that there was no significant $O_{2}^{\cdot -}$ signal (blue color) after one week of DMTU treatment. In contrast, a strong $O_{2}^{\cdot -}$ signal could be detected in the entire IM domain (Figure 8C,D). It has been reported that $O_{2}^{\cdot -}$ accumulation can maintain *WUS* expression and that the delicate Arabidopsis seedlings cannot generate SAM [10]. Given this, we tried to understand the role of $O_{2}^{\cdot -}$ in regulating *WUS* expression in proliferative IM. The longitudinal section results of the *proWUS::GUS* line revealed that after DMTU treatment, the *WUS* expression signal disappeared in the IM at 2 WAB (Figure 8F), which should show the *WUS* expression signal (Figure 8E, mock), suggesting that endogenous $O_{2}^{\cdot -}$ accumulation could also regulate *WUS* expression in proliferative IM.

**Figure 9:**
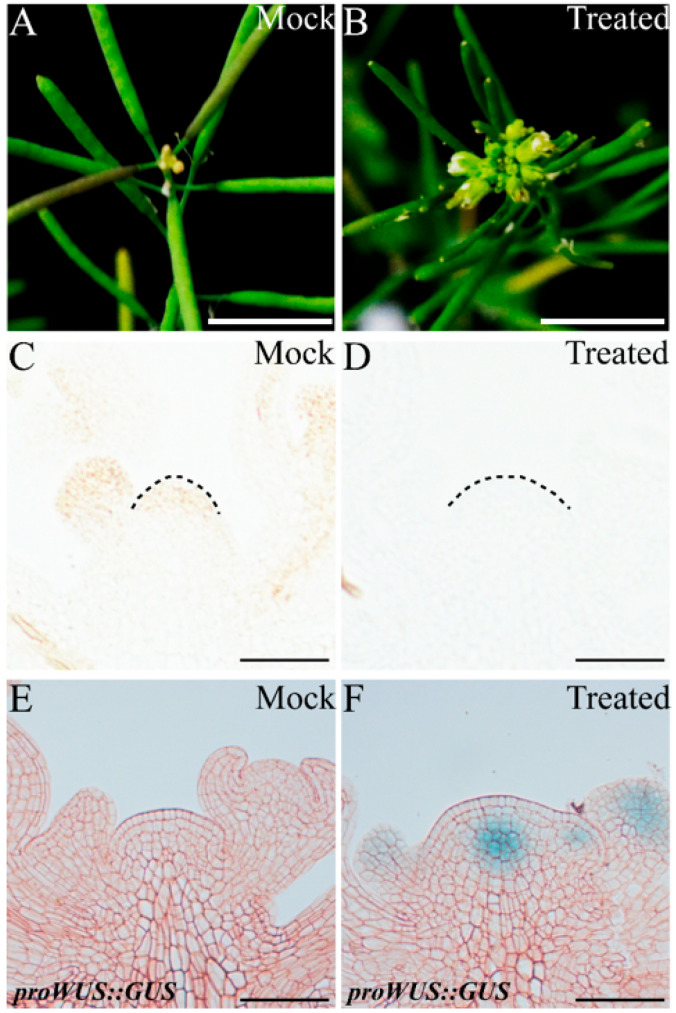
The phenotype of primary shoot inflorescence (**A**,**B**), H_2_O_2_ accumulation (**C**,**D**), and *WUS* expression profile (**E**,**F**) of the control group (mock) and 5 mM KI-treated group. Black dotted lines indicate the shape of the IM. Scale bars: 1 cm in (**A**,**B**), 35 μm in (**C**–**F**).

It has been reported that H_2_O_2_ accumulation can repress *WUS* expression in vegetative SAM [10] and possibly inhibit *WUS* expression in proliferative IM [2]. Next, we wondered whether endogenous H_2_O_2_ accumulation restrains *WUS* expression in proliferative IM. At 3 WAB, the inflorescence of the control group (mock) displayed a flowering-arrested phenotype (Figure 9A). However, IM treated with 5 mM KI for one week showed relatively high activity due to several opening flowers surrounding the IM (Figure 9B). Then, we monitored H_2_O_2_ accumulation in control and KI-treated IMs. Compared with the control IM, which showed clear H_2_O_2_ accumulation in the stem cell population, the KI-treated IM displayed an undetectable H_2_O_2_ signal (brown color), indicating that 5 mM KI could eliminate endogenous H_2_O_2_ efficaciously in IM (Figure 9C,D). Furthermore, we found that *WUS* was certainly transcribed in the KI-treated IM but terminated in the control IM (Figure 9E,F). These results suggested that endogenous H_2_O_2_ accumulation in proliferative IM could repress *WUS* expression.

### 2.5. The Expression Profiles of ROS Metabolism-Related Genes

**Figure 10:**
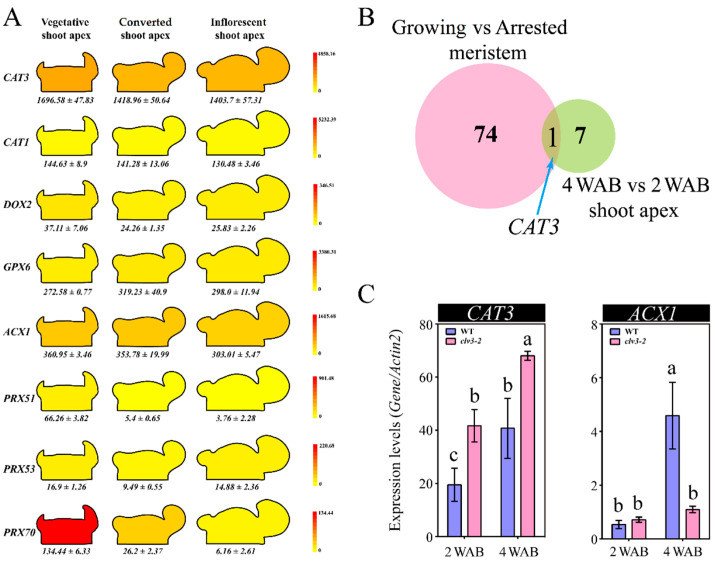
The expression profiles of ROS metabolism-related genes. (**A**) The expression patterns of eight ROS metabolism-related genes in the BAR database. The gene expression data in vegetative, converted (i.e., the transition from the vegetative stage to the proliferative stage), and inflorescent (i.e., proliferative stage) shoot apex are shown. (**B**) Common gene isolation between two published RNA-seq datasets. One is the comparative data using growing and arrested meristems, and the other is the comparative data using 2 WAB and 4 WAB shoot apexes. (**C**) The expression profiles of the ROS clearance-related gene *CAT3* and ROS production-related *ACX1* at 2 WAB and 4 WAB in WT and *clv3-2* IM tissues. qRT–PCR assays were performed. Each experiment was replicated three times, and error bars indicate SD. One-way ANOVA post Tukey’s HSD test (*p* < 0.05) was carried out to calculate the differences among different groups. Different letters indicate significant differences, while the same letters indicate no significant differences.

In our previous work, a total of eight ROS metabolism-related factors, including two catalase (CAT)-encoding genes, three peroxidase (PRX)-encoding genes (*PRX51*, *PRX53*, and *PRX70*), one α-dioxygenase (DOX2)-encoding gene, one glutathione peroxidase 6 (GPX6)-encoding gene, and one acyl-CoA oxidase 1 (ACX1)-encoding gene, were identified by RNA sequencing [2]. In this study, we further submitted these candidate genes to the BAR database to check their expression patterns in shoot apexes at vegetative, converted (i.e., the transition from the vegetative stage to the proliferative stage), and inflorescent (i.e., proliferative stage) stages. The results showed that only *CAT3* and *ACX1*, which have opposite roles in H_2_O_2_ metabolism, were highly expressed in all three types of shoot apex tissues (Figure 10A), indicating that they might play roles in regulating shoot apex development. In addition, we compared our previous RNA-sequencing data (4 WAB vs. 2 WAB, shoot apex) [2] with the published transcriptome data of growing and arrested shoot meristem [19]. Only *CAT3* was identified as a common gene in the overlap between 75 and 8 ROS metabolism-related genes (Figure 10B). To understand the internal expression profiles of *CAT3* and *ACX1* in WT and *clv3-2* IM tissues, we carried out a quantitative real-time PCR (qRT–PCR) assay using IM tissues at 2 WAB and 4 WAB. The results indicated that *CAT3* was upregulated at 4 WAB in both WT and *clv3-2* IMs compared with that at 2 WAB, but the expression levels in *clv3-2* IM were significantly increased at 4 WAB compared with that in WT. In addition, *ACX1* was significantly induced at 4 WAB in WT IM, while there was no change in *clv3-2* IM at the same time point (Figure 10C). These results revealed that *CAT3* and *ACX1* might take part in H_2_O_2_ metabolism during shoot apex development and that the *clv3-2* mutant might have a higher ability of H_2_O_2_ clearance than WT.

## 3. Discussion

Senescence and death are inevitable processes for tissues, organs, somatic cells, and even stem cells. In Arabidopsis, WUS plays essential roles in governing shoot and floral meristem identity, reproductive organ development, and embryogenesis [20,21]. Therefore, the termination of *WUS* expression in IM is the precondition for stem cells entering senescence and death processes [2]. Given that *WUS* directly determines stem cell activity and longevity and thus affects plant lifespan, it is necessary to determine the age-dependent repressor of *WUS*. Although the preceding evidence gives us some cues [2,10], the relationships among stem cell marker genes, ROS components, and age-dependent dPCD during stem cell senescence and death processes are still largely unknown. In this study, our results mainly reveal the possible linkage between stem cell marker genes and ROS metabolism in aging stem cells undergoing dPCD.

### 3.1. Dynamic Changes in ROS Components May Play Key Roles in Controlling Stem Cell Longevity in Arabidopsis thaliana

It is well known that the WUS-CLV3 negative feedback loop regulates stem cell activity and maintenance [22,23]. To date, several studies have proven that the WUS-CLV3 negative feedback loop can be modulated by different factors [7]. Among these factors, the ROS signaling pathway plays an essential role in regulating the stem cell population. For instance, loss of function of the mitochondrial protease AtFTSH4 results in the abnormal accumulation of ROS in the shoot apex and therefore induces SAM termination [24]. These findings indicate the regulatory link between the WUS-CLV3 feedback loop and ROS homeostasis in controlling stem cell development during the vegetative and proliferative stages of Arabidopsis.

**Figure 11:**
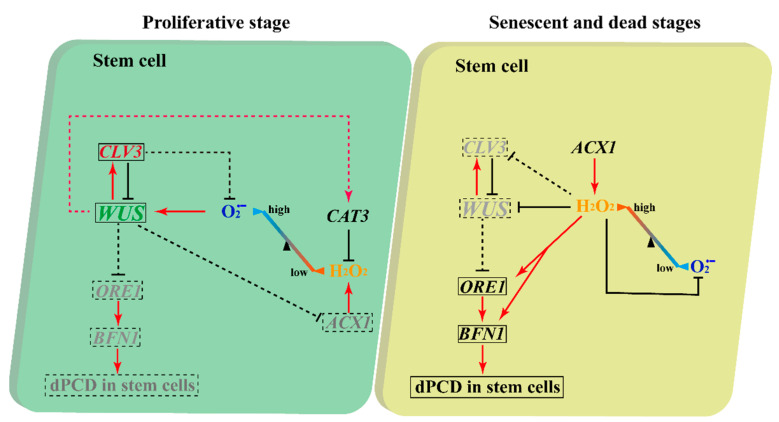
The putative model of ROS-mediated dPCD in stem cells. During the proliferative stage, *WUS* is promoted by $O_{2}^{\cdot -}$ and inhibited by *CLV3* at the same time. *WUS* may promote *CAT3* expression and repress *ACX1* expression to keep $O_{2}^{\cdot -}$ at high levels and H_2_O_2_ at low levels in stem cells. Meanwhile, *WUS* may repress the *ORE1*-*BFN1* cascade to prevent dPCD from occurring at the wrong time point. *CLV3* may also inhibit $O_{2}^{\cdot -}$ production via unknown pathways to repress *WUS* indirectly. During senescence and death stages, *ACX1* may induce age-dependent H_2_O_2_ accumulation and bursts in stem cell populations. A high level of H_2_O_2_ can decrease $O_{2}^{\cdot -}$ production and terminate *WUS* directly. Furthermore, the H_2_O_2_ burst may repress *CLV3*, leading to the full termination of the WUS-CLV3 feedback loop. Moreover, H_2_O_2_ directly triggers the dPCD process by activating the *ORE1-BFN1* cascade in stem cells. Lines and arrows indicate the known processes. Dotted lines and arrow lines denote the putative processes.

During the proliferative, senescent, and dying stages of stem cells, we found that a dynamic conversion of ROS components from $O_{2}^{\cdot -}$ to H_2_O_2_ was carried out in both WT and *clv3-2* mutants but in a different time series (Figure 1 and Figure 2). The *WUS* expression period fully overlapped with the time window of detection of $O_{2}^{\cdot -}$ in WT and *clv3-2* mutants (Figure 1 and Figure S2). More importantly, the WT IM showed a precocious flowering-arrested phenotype and *WUS* expression termination after endogenous $O_{2}^{\cdot -}$ was removed by the scavenger DMTU (Figure 8). These results revealed that $O_{2}^{\cdot -}$ can promote *WUS* expression during stem cell aging processes. It has been reported that another ROS component, H_2_O_2_, accumulates in PZ and inhibits the production of $O_{2}^{\cdot -}$ during stem cell development [10]. Our data indicate that the accumulation of H_2_O_2_ in the SAM domain inhibited the production of $O_{2}^{\cdot -}$ during the conversion stage. Thus, the spatial balance between $O_{2}^{\cdot -}$ and H_2_O_2_ in the shoot apex is applicable to temporal ROS dynamics in stem cells. In addition, the exogenous H_2_O_2_ assay demonstrated that H_2_O_2_ can rapidly repress *WUS* expression (Figure 6). In contrast, the KI-treated IM maintained prolonged *WUS* activity, leading to prolonged longevity of stem cell populations (Figure 9). These results suggested that endogenous H_2_O_2_ accumulation in aging IM suppressed *WUS* expression via an unknown straightforward pathway. Moreover, we previously found that the expression time of *CLV3* was 1 week longer than *WUS* [2] and partially overlapped the H_2_O_2_ detection time (Figure 2), suggesting that H_2_O_2_ accumulation might inhibit *CLV3* expression after *WUS* termination. In contrast, we found that the higher levels of exogenous H_2_O_2_ enhanced the expression of the dPCD marker genes *ORE1* and *BFN1* in stem cell layers, indicating that the accumulation and burst of endogenous H_2_O_2_ may directly induce dPCD in the stem cell population in a WUS-independent pathway (Figure 2, Figure 3, Figure 4 and Figure 5). Based on these results, we hypothesized that dynamic changes in endogenous $O_{2}^{\cdot -}$ and H_2_O_2_ not only shut down the proliferative activity of IM but also activated dPCD in the stem cell population. The dynamic shift from $O_{2}^{\cdot -}$ to H_2_O_2_ might play a key role in governing stem cell longevity (Figure 11).

### 3.2. The Essential Roles of Stem Cell Marker Genes in Regulating Stem Cell Fate in Arabidopsis during the Proliferative Stage

Clearly, *ACX1* is involved in the production of H_2_O_2_ [25]. In contrast, *CAT3* catalyzes the decomposition of H_2_O_2_ and plays a critical role in controlling the homeostasis of ROS in Arabidopsis [26]. When *WUS* was expressed in the IM domain, there were no detectable H_2_O_2_ signals in the IM domain, especially in stem cell layers in WT and *clv3-2* mutants. Interestingly, weak H_2_O_2_ signals started to be observed in the stem layers of the WT and IM domains of *clv3-2* mutants when *WUS* expression was terminated at 4 WAB in WT and 7 WAB in *clv3-2* mutants (Figure S2). This evidence indicates that *WUS* may inhibit H_2_O_2_ production through the regulation of the H_2_O_2_ biosynthetic gene *ACX1*. Consistent with this, the relative expression level of *ACX1* at 4 WAB, when *WUS* was not expressed, was maximally 6-fold compared to that at 2 WAB, when *WUS* was expressed (Figure 10C and Figure S2). In addition, *WUS* may also promote *CAT3* to eliminate H_2_O_2_ as a homeostatic regulation, resulting in a low concentration of H_2_O_2_ in stem cells. Consistently, the relative expression level of *CAT3* at 4 WAB was significantly higher than that at 2 WAB due to the prolonged *WUS* expression period in *clv3-2* mutants (Figure 10C and Figure S2). However, we found that the relative expression intensity of *CAT3* at 4 WAB was also notably higher than that at 2 WAB in WT but lower than that at 4 WAB in *clv3-2* mutants (Figure 10C), indicating that there may be some unknown signals inducing *CAT3* expression. Thus, we thought that *WUS* might regulate the dynamic changes in ROS via the transcriptional regulatory pathway. Moreover, we thought that the dynamic changes in ROS might control *WUS* expression (see ‘Discussion’ Section 3.1). Based on these findings, we speculated that *WUS-*, *CAT3-*, and *ACX1*-mediated ROS changes formed a feedback pathway to regulate stem cell fate during the proliferative stage (Figure 11).

In addition, the spatial-temporal expression levels of *WUS* and the dPCD marker genes *ORE1* and *BFN1* also reflect their potential regulatory relationship. In WT, *WUS* was expressed in the organizing center (OC) at 1 WAB and 2 WAB and was terminated at 3 WAB (Figure S2) [2]. The dPCD marker genes *ORE1* and *BFN1* displayed quite similar expression profiles. Both genes were initially expressed in the Rib zone in WT and *clv3-2* mutants. The appearance of dPCD marker genes in stem cell layers was 1 week later than *WUS* termination in both WT and *clv3-2* mutants (Figure 4, Figure 5 and Figure S2). These results suggested that the expression of *WUS* might indirectly but strictly restrict the activities of dPCD marker genes in the IM domain, thereby protecting the stem cell population to prevent the dPCD process from untimely activation during the proliferative stage in Arabidopsis. Furthermore, the results indicated that the possible functions of another stem cell marker gene, *CLV3,* may restrict the production of $O_{2}^{\cdot -}$. Compared with WT, the existence time of $O_{2}^{\cdot -}$ is prolonged in *clv3-2* mutants (Figure 1). In addition, *CLV3* can repress *WUS* expression, but how CLV3 signaling leads to *WUS* repression is still unclear. Our previous study revealed that *CLV3* expression time is sustained until 4 WAB, while the expression of *WUS* was terminated at 3 WAB [2], and that loss-of-function of *CLV3* prolonged the expression time of *WUS* (Figure S2) [2]. Combining these findings, it is highly possible that *CLV3* may prevent *WUS* reactivation via the inhibition of the $O_{2}^{\cdot -}$ metabolic pathway (Figure 11).

## 4. Materials and Methods

### 4.1. Plant Materials and Growth Conditions

In this study, *Arabidopsis thaliana* in the Landsberg *erecta* (L*er*) background (wild-type) was used as the plant material. In addition, *clv3-2* mutant (L*er*) was also used as the plant material and has already been described in the previous study [27]. Besides, the WUS promotor reporter lines *proWUS::GFP-ER* and *proWUS::GUS* were used to detect the expression pattern of *WUS* and have been reported previously [2]. The *proBFN1::GUS-GFP* [2] and *proORE1::GUS-GFP* (see Section 4.6) in the *clv3-2* background were generated by crossing with the *clv3-2* mutant line. Arabidopsis seeds were sown in pots containing vermiculite and nutrition soil (Baseconnect, Osaka, Japan) and incubated at 4 °C in the dark for 3 days to promote germination. All plants were cultured in an illumination incubator (BiOTRON, LPH-411SP, Osaka, Japan) under a 16-h light (100 µmol m^−2^ s^−1^)/8-h dark light cycle with 60% humidity at 22 °C.

### 4.2. Phenotypic Definitions and Measurements

In our previous study, we had established a time system to observe the development of each Arabidopsis plant precisely. The term “weeks after bolting” (WAB) was used as the temporal unit [28]. The stem length was measured every day until it approached 1 cm. We defined this time point as the beginning of bolting [29]. In order to count the number of flowers on the main shoot, siliques and flowers (older than stage 7) were counted at the same time. The developmental stage of flower was described previously [30]. Morphological observations of inflorescences on main shoots were photographed using a Canon EOS 600D optical camera (Canon, Tokyo, Japan).

### 4.3. GUS Staining and Tissue Sectioning

For tissues sectioning, inflorescences of main shoots of promotor reporter lines were cut off without any dissection and fixed in 90% acetone solution for 15 min at room temperature. Subsequently, the tissues were rinsed with double-distilled water, and then stained using GUS staining solution. The GUS staining method was previously described by Shirakawa et al. [31]. Tissue sectioning method was described by Yamaguchi et al. previously [32]. The slides were stained with 0.05% neutral red (Wako Chemicals, Kyoto, Japan) for 5 min and rinsed with double-distilled water.

### 4.4. Confocal Microscopy

The IMs on *proWUS::GFP-ER* primary shoots were used to observe the GFP signals. The floral buds on inflorescences older than stage 7 were cut off with tweezers under a light microscope. The IMs were embedded into 5% agar (Wako Chemicals, Kyoto, Japan) and sliced with a Liner Slicer PRO7 vibratome (Dosaka, Kyoto, Japan) [32]. The resulting tissue sections were immersed in moderate volumes of 1/10 Murashige and Skoog (MS) [33] solution on glass slides. The GFP signal was immediately observed under an FV 1000 (Olympus, Tokyo, Japan) microscope with FV10-ASW software (https://www.olympus-lifescience.com.cn/en/support/downloads/#!dlOpen=%23detail847249651 (accessed on 7 March 2022); see Laser Scanning Microscopes option). GFP was excited using the 488-nm argon laser and its emission signal was monitored between 495 and 545 nm.

### 4.5. Reverse-Transcription PCR and Quantitative Real-Time PCR (qRT–PCR)

For the extraction of total RNA, the RNeasy Plant Mini Kit (Qiagen, Hilden, Germany) was used. To prevent the DNA contamination, the RNase-Free DNase Set (Qiagen, Hilden, Germany) was used to digest the genomic DNA in the RNA samples. Reverse-transcription PCR was carried out using PrimeScript™ RT Master Mix (Takara, Shiga, Japan). The method of qRT–PCR was described previously [32]. Arabidopsis *ACTIN2* (*AT3G18780*) [34] was used as the internal reference. The relative expression levels of genes were showed the ratio of gene to *ACTIN2*. Each qRT-PCR experiment was performed three times repeatedly. The primers used are listed in Table S1.

### 4.6. Plasmid Construction and Plant Transformation

The *proBFN1::GUS-GFP* transgenic line was reported previously [2]. The *proORE1::GUS-GFP* line (2.5 kb promoter) was generated using the same method mentioned previously [2]. The *Agrobacterium*-mediated floral dip method [35] was carried out to generate transgenic plants. T1 seeds were collected and screened using the chemical Basta (Wako Chemicals, Kyoto, Japan). More than 20 T1 plants were obtained, and the representative lines were chosen for further study. The primers used are listed in Table S1.

### 4.7. DAB and NBT Staining

DAB (3,3′-diaminobenzidine, Sigma–Aldrich, Tokyo, Japan) and NBT (4-nitro blue tetrazolium chloride, Sigma–Aldrich, Tokyo, Japan) staining of IM was performed using methods described previously [10]. The chlorophyll in stained IM tissues was discolored in boiling ethanol (ethanol:glycerin:glacial acetic acid = 3:3:1). For each experiment, at least 5 individual inflorescences were stained.

### 4.8. FDA and PI Staining

FDA and PI staining methods were described previously [2]. Confocal microscope observation was performed using previously published methods [2].

### 4.9. Exogenous H_2_O_2_ and ROS Scavenger Treatments

For exogenous H_2_O_2_ treatment in the *proWUS::GUS* line, 1 WAB-old primary shoot inflorescences were immersed in a 5, 10, or 20 mM H_2_O_2_ (WAKENYAKU, Shiga, Japan) solution containing 0.01% Silwet L-77 for 10 s each day. The treatment was maintained for one week. After confirmation of the optimal concentration, primary shoot inflorescences of the *proWUS::GUS* line at 1 WAB were immersed in a 5 mM H_2_O_2_ solution to treat for 1, 2, 3, 4, 5, and 6 days to confirm the response speed of *WUS*. For exogenous H_2_O_2_ treatment in *proORE1::GUS-GFP* and *proBFN1::GUS-GFP* lines, primary shoot inflorescences at 2 WAB were immersed in a 5, 20, 40, or 50 mM H_2_O_2_ solution for 10 s each day. The treatment was maintained for one week. For the control group, double distilled water containing 0.01% Silwet L-77 was used. For each experiment, at least 5 individual inflorescences were treated.

For ROS scavenger treatment in the WT and *proWUS::GUS* lines, primary shoot inflorescences at 1 WAB were immersed in a 20 mM DMTU (Sigma–Aldrich, Tokyo, Japan) solution containing 0.01% Silwet L-77 for 10 s each day, and primary shoot inflorescences at 2 WAB were immersed in a 5 mM KI (Sigma–Aldrich, Tokyo, Japan) solution containing 0.01% Silwet L-77 for 10 s each day. The treatment was maintained for one week. For the control group, double distilled water containing 0.01% Silwet L-77 was used. For each experiment, at least 5 individual inflorescences were treated.

### 4.10. Data Statistics and Availability

One-way ANOVA post Tukey’s HSD test (*p* < 0.05) was carried out to calculate the differences among different groups. Different letters indicate significant differences, while the same letters indicate no significant differences. RNA-seq datasets were downloaded from the DNA Data Bank of Japan (DDBJ) with the accession number DRA010789 [2] and from the National Center for Biotechnology Information GEO with the accession numbers GSE74386 and GSE79287 [19]. The expression patterns of 8 ROS-related genes were obtained from the BAR database (http://bar.utoronto.ca/efp/cgi-bin/efpWeb.cgi) (accessed on 7 March 2022).

## 5. Conclusions

In this study, we examined the dynamic changes in the ROS components $O_{2}^{\cdot -}$ and H_2_O_2_ in the stem cell population and revealed their potential regulatory relationship between stem cell marker genes using morphological and physiological methods. Based on the results of this study, we established an ROS-mediated regulatory model of the age-dependent dPCD process during stem cell death. In our putative model, the WUS-CLV3 feedback loop was the regulatory core: at the proliferative stage, the high level of $O_{2}^{\cdot -}$ maintained *WUS* expression, while *CLV3* repressed *WUS* expression at the same time. The expression of *WUS* not only prevented the activation of the *ORE1-BFN1* cascade, thereby avoiding dPCD in the stem cell population, but also might promote *CAT3* and repress *ACX1* to maintain a low level of H_2_O_2_. In addition, it was possible that *CLV3* inhibited $O_{2}^{\cdot -}$ production. Along with the growth of Arabidopsis, the shoot stem cells started to enter the senescent and dead stages, and H_2_O_2_ inevitably and significantly accumulated in IM. A high level of H_2_O_2_ further suppressed $O_{2}^{\cdot -}$ production, resulting in $O_{2}^{\cdot -}$ becoming undetectable in IM. Because the WUS-CLV3 loop was ultimately collapsed by high levels of H_2_O_2_, the *ORE1-BNF1* cascade was activated, leading to dPCD in the stem cell population. Notably, the promotion of *ACX1* might be a key factor in H_2_O_2_ accumulation in IM (Figure 11). More molecular and genetic studies mainly focusing on *CAT3* and *ACX1* should be performed in the future to reveal the ROS-mediated mechanism of stem cell death.

## Data Availability

Not applicable.

## Supplementary Materials

The following are available online at https://www.mdpi.com/article/10.3390/ijms23073864/s1.
