# Supplementary material for: Dynamic Changes in Reactive Oxygen Species in the Shoot Apex Contribute to Stem Cell Death in Arabidopsis thaliana
Source: Int J Mol Sci. 2022 Mar 31;23(7):3864. doi: 10.3390/ijms23073864 (PMC8998593; doi:10.3390/ijms23073864)
Supplement: Supplementary file 1 [file ijms-23-03864-s001.zip › Supplementary Figures.pdf]

Supplementary Figures:

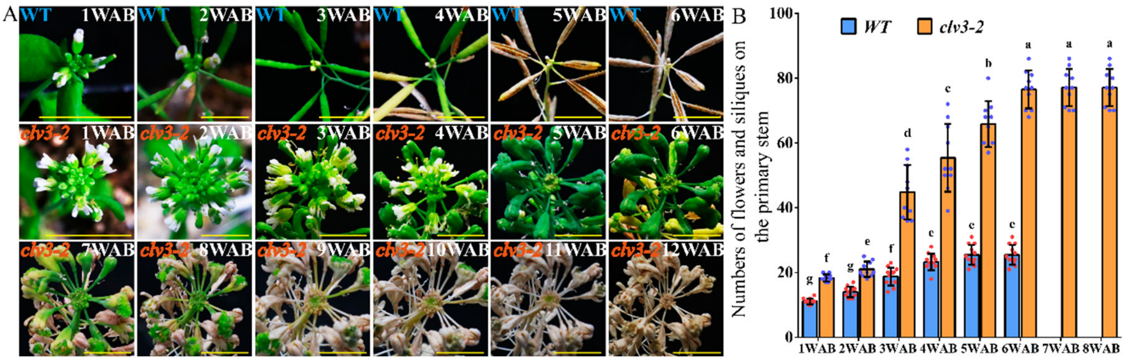

Figure S1. The *clv3-2* mutant showed a prolonged lifespan of the IM. (**A**) Morphological changes in WT and *clv3-2* inflorescences. Scale bars = 1 cm. (**B**) The statistical analysis of the main shoot flower and silique numbers in the WT and *clv3-2* mutant. One-way ANOVA post Tukey's HSD test ($p < 0.05$) was carried out to calculate the differences among different groups. Over 10 individual seedlings of WT and *clv3-2* were observed. Different letters indicate significant differences, while the same letters indicate no significant differences.

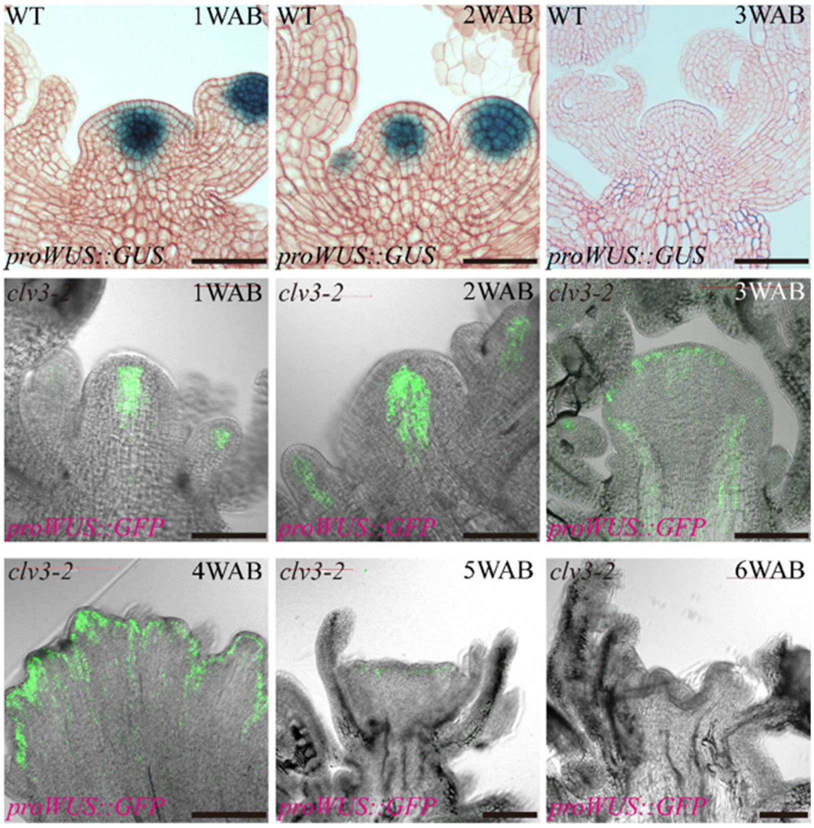

Figure S2. Spatial-temporal expression patterns of *WUS* in IMs of WT and *clv3-2* mutants. The *proWUS::GUS* and *proWUS::GFP-ER* reporter lines (in the *clv3-2* background) were used. Scale bars: 25 μm in WT and 100 μm in *clv3-2* mutant.

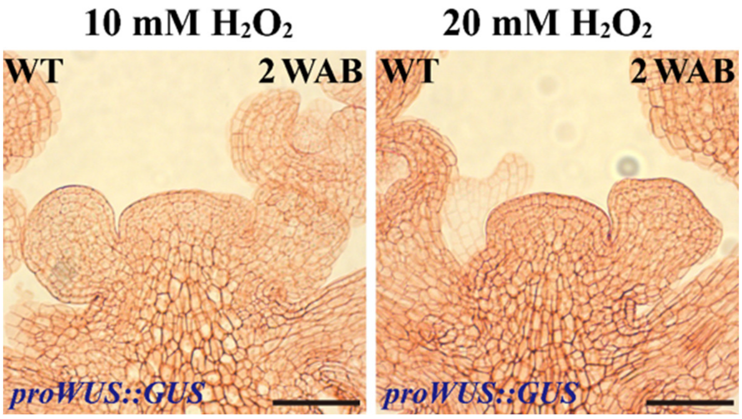

Figure S3. Different concentrations of exogenous H_2O_2 inhibited *WUS* expression in WT IMs after one week of continuous treatment. Scale bars = 30 μm.

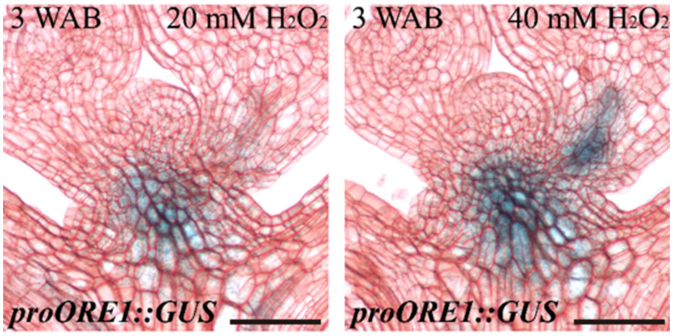

Figure S4. Treatment with 20 and 40 mM exogenous H_2O_2 did not change the *ORE1* expression pattern in WT IMs after one week of continuous treatment. Scale bars = 30 μm.
