# Supplementary material for: Dynamic Changes in Reactive Oxygen Species in the Shoot Apex Contribute to Stem Cell Death in Arabidopsis thaliana
Source: Int J Mol Sci. 2022 Mar 31;23(7):3864. doi: 10.3390/ijms23073864 (PMC8998593; doi:10.3390/ijms23073864)
Supplement: Supplementary file 1 [file ijms-23-03864-s001.zip › Table S1.pdf]

Table S1. The primers used in this study.

Gene Name	Forward (5′-3′)	Reverse (5′-3′)	Annotation
proORE1	CACCGAGCCAGAAAACGGTCTTTGGGTAA	TGCCTCGTAATCCATTTTATCCTA	Cloning of 2.5kb promoter
CAT3	CTTGTGGTTCCTGGAATCTACT	AGGATCAAACTTTGAGGGGTAG	Gene validation by using Real time PCR
ACX1	GAGGATATGAAGATCGTCTGGG	TCATTGAGACGAAGCTCGATAA	
ACTIN2	GAAAAGATCTGGCATCACACTTTATA	ACATACATAGCGGGAGAGTTAAAGGT	Internal reference
